# Prevalence of molecular markers of drug resistance in an area of seasonal malaria chemoprevention in children in Senegal

**DOI:** 10.1186/1475-2875-12-137

**Published:** 2013-04-23

**Authors:** Aminata C Lo, Babacar Faye, El-Hadj Ba, Badara Cisse, Roger Tine, Annie Abiola, Magatte Ndiaye, Jean LA Ndiaye, Daouda Ndiaye, Cheikh Sokhna, Jules F Gomis, Yemou Dieng, Omar Faye, Omar Ndir, Paul Milligan, Matthew Cairns, Rachel Hallett, Colin Sutherland, Oumar Gaye

**Affiliations:** 1 Service of Parasitology, Faculty of Medicine, University Cheikh Anta Diop, Dakar, Senegal; 2 London School of Hygiene and Tropical Medicine, London, UK; 3 Institut de Recherche pour le Développement, Dakar, Senegal

**Keywords:** Plasmodium falciparum, SMC, Sulphadoxine-pyrimethamine, Amodiaquine, Prevalence, Pfdhfr, Pfdhps, Pfcrt, Pfmdr1

## Abstract

**Background:**

In sub-Saharan Africa, malaria is the leading cause of morbidity and mortality especially in children. In Senegal, seasonal malaria chemoprevention (SMC) previously referred to as intermittent preventive treatment in children (IPTc) is a new strategy for malaria control in areas of high seasonal transmission. An effectiveness study of SMC, using sulphadoxine-pyrimethamine (SP) plus amodiaquine (AQ), was conducted in central Senegal from 2008 to 2010 to obtain information about safety, feasibility of delivery, and cost effectiveness of SMC. Here are report the effect of SMC delivery on the prevalence of markers of resistance to SP and AQ.

**Methods:**

This study was conducted in three health districts in Senegal with 54 health posts with a gradual introduction of SMC. Three administrations of the combination AQ + SP were made during the months of September, October and November of each year in children aged less than 10 years living in the area. Children were surveyed in December of each year and samples (filter paper and thick films) were made in 2008, 2009 and 2010. The prevalence of mutations in the *pfdhfr, pfdhps, pfmdr1* and *pfcrt* genes was investigated by sequencing and RTPCR in samples positive by microscopy for *Plasmodium falciparum*.

**Results:**

Mutations at codon 540 of *pfdhps* and codon 164 of *pfdhfr* were not detected in the study. Among children with parasitaemia at the end of the transmission seasons, the CVIET haplotypes of *pfcrt* and the 86Y polymorphism of *pfmdr1* were more common among those that had received SMC, but the number of infections detected was very low and confidence intervals were wide. The overall prevalence of these mutations was lower in SMC areas than in control areas, reflecting the lower prevalence of parasitaemia in areas where SMC was delivered.

**Conclusion:**

The sensitivity of *P. falciparum* to SMC drugs should be regularly monitored in areas deploying this intervention. Overall the prevalence of genotypes associated with resistance to either SP or AQ was lower in SMC areas due to the reduced number of parasitaemia individuals.

## Background

In many parts of sub-Saharan Africa, malaria remains the leading cause of morbidity and mortality in children. Malaria caused an estimated 216 million cases of clinical malaria and 655 thousand deaths in 2010 [1]. In areas of seasonal malaria transmission, the burden of severe disease and mortality due to malaria is mainly among children under five years of age. Seasonal malaria chemoprevention (SMC) previously referred to as intermittent preventive treatment in children (IPTc), is a new control strategy suited to areas of high seasonal transmission. SMC is defined as the intermittent administration of full treatment of an anti-malarial medicine during the malaria season to prevent malarial illness with the objective of maintaining therapeutic anti-malarial drug concentrations in the blood throughout the period of greatest malarial risk [2].The World Health Organization now recommends that children living in areas of high seasonal transmission should receive treatment with sulphadoxine-pyrimethamine (SP) plus amodiaquine (AQ) each month for up to four months during the peak transmission period. SP and AQ currently retain their efficacy in areas where SMC is recommended but it is important that sensitivity of parasites to SMC drugs is monitored in areas where it is introduced.

The presence of *Plasmodium falciparum* dihydrofolate reductase (*pfdhfr*) triple mutation (codon 108, 51 and 59) together with double *P. falciparum* dihydropteorate synthetase (*pfdhps*) mutations at codons 437 and 540 is associated with resistance to SP [3,4]. In West Africa, the mutation at codon 540 of the *pfdhps* is very rare and its presence is a useful single marker of quintuple mutation (triple *pfdhfr* plus double *pfdhps*). In addition to markers of resistance to SP, mutations at *P. falciparum* chloroquine transporter (*pfcrt*) and *P. falciparum* multidrug resistance (*pfmdr1*) genes should be assessed to monitor SP+AQ efficacy in areas where SMC is implemented. The *pfcrt* mutant haplotype (CVIET) based on codon 72–76, which mediates high levels of resistance to chloroquine (CQ), has been linked with moderate resistance to AQ in Africa in the presence of particular haplotypes of p*fmdr1*[5,6]. IPTi in infants in Senegal with SP revealed that despite an increase in the prevalence of individual mutations, there was no major impact on dihydrofolate reductase (*pfdhfr*) and dihydropteroate synthetase (*pfdhps*) combined mutations [7].

In this study, the prevalence of molecular markers of *pfcrt* and *pfmdr1* was determined to evaluate the effectiveness of the use of AQ, as in several studies in Mali [8,9]. The aim of this study was to assess the prevalence of molecular markers of resistance to SP (*pfdhfr* and *pfdhps*) and AQ (*pfcrt* and *pfmdr1*) in an area where SMC using both drugs was being implemented at large scale.

## Methods

### Study site and subjects

According to the 2002 national census, approximately 450,000 people live in this area. The northern part of the study area is part of the district medical Bambey while the southern part of the district medical Fatick. There are approximately 725 villages in the study area and 54 health posts run by health workers and two health centers directed by a medical doctor.

The study site and the implementation of SMC are described by Cisse *et al.*[10]. In brief, SMC with SP+AQ was administered once per month from September to November to children in three districts in central Senegal (Mbour, Bambey and Fatick) from 2008 to 2010. Implementation of SMC was phased in the 54 health posts. Nine health posts delivered SMC in 2008, 27 health posts in 2009, and 45 in 2010. Initially children aged three to 59 months were included in the programme; in 2009 the upper age limit was increased to include all children less than 10 years of age.

At the end of each transmission season, a cross-sectional survey of children was conducted to measure the prevalence of parasitaemia and to take blood samples on filter paper for analysis of molecular markers of resistance to SMC drugs. In 2008, the population in the catchment of 27 health posts was surveyed, including the nine health posts that delivered SMC, and 18 control health posts that did not deliver SMC. In 2009 and 2010, all 54 health posts were included in the survey. Sampling was stratified by health post area, with probably sampling of individuals within each stratum.

For each child signed consent was obtained from a parent or guardian after explaining the aims and procedures of the survey. Filter paper samples from individuals who had a positive blood film for *P. falciparum* were used to determine the presence of mutations in genes *pfdhfr, pfdhps, pfmdr1* and *pfcrt* associated with resistance to SP and to AQ by sequencing and Real Time PCR, to measure the impact of SMC on the prevalence of these mutations at the end of each transmission season.

### Ethical approval

The study was approved by the Ethics Committees of the Ministry of Health, Senegal and the London School of Hygiene & Tropical Medicine.

### Laboratory methods

#### Microscopy

Thick smears were stored at room temperature for 48 hours, washed with pierval water, pH 7.2 and stained in a 6% Giemsa solution for 20 minutes. Microscopy was used to detect absence or presence of asexual and sexual parasites. Total number of parasites seen and total number of white blood cells (WBCs) counted were used to work out parasitaemia per μl assuming that 1 μl contains 8,000 WBCs. Quality control was performed by reading 10% of all slides by a different experienced laboratory technician; in case of discrepancies a third reading was performed and the average results obtained with the two closest.

#### DNA extraction

The genomic DNA of the parasite was extracted from filter papers reported positive after microscopic examination using the chelex method according to methods described by Plowe *et al.*[11] and K76T was determined in at least two multiplex real-time PCR runs using the Rotorgene 3000 platform previously described [12]. 3D7, Dd2 and 7G8 DNA obtained from the Malaria Research Reagent Resource (MR4) were used to provide sequence-specific positive control.

#### Genotyping

The genotype of the *pfcrt* gene for polymorphisms C72S and K76T was determined in at least two multiplex real-time PCR runs using the Rotorgene 3000 platform previously described [8]. 3D7, Dd2 and 7G8 DNA obtained from the Malaria Research Reagent Resource (MR4) were used to provide sequence-specific positive control.

Amplification of *pfdhfr, pfdhps* and *pfmdr1* genes involved primers is described in Table 1. All amplicons of the *pfdhfr*, *pfdhps* and *pfmdr1* genes were re-amplified on a nested PCR step. Amplicons from nested PCR reaction were purified using the QIA quick PCR Purification Kit according to manufacturer’s instructions and subjected to di-deoxy fluorescent sequencing (Big Dye 3.1) using conditions and sequencing primers pairs. The sequence of amplified DNA products was determined using ABI PRISM Genetic Analyser. Chromas software was used to analyse the sequence results. The DNA sequence was compared with reference sequence of the *pfdhfr, pfdhps* and *pfmdr1* portions of the *P. falciparum* 3D7 clone using Blast similar alignment. Primers sequences are summarized in Table 1.

**Table 1:** ***Pfdhfr*, *pfdhps*, *pfmdr1 *and *pfcrt *PCR primers sequences used in amplification reactions**

**Genes names**	**Primers names**	**Primers sequences**	**amplicon size (bp)**	**PCR cycling conditions**
*Pfdhfr*				
PCR1	dhfr_M1	5′-TTTATGATGGAACAAGTCTGC-3′	650	93°C for 5min/(94°C for 30s- 54°C for 60s- 65°C for 60s) × 41cycles/ 65° for 5min/ 15°C for 5min
Nested	dhfr_M7	5′-CTAGTATATACATCGCTAACA-3′		
	dhfr-M9	5′-CTGGAAAAAATACATCACATTCATATG-3′	594	95°C for 5min/(93°C for 30s- 56°C for 30s- 68°C for 75s) × 30 cycles/ 75°C for 5min
Sequencing	dhfr-M3	5′-TGATGGAACAAGTCTGCGACGTT-3′		
	dhfr-M9	5′-CTGGAAAAAATACATCACATTCATATG-3′		96°C for 1min/(96°C for 30s- 50°C for 30s- 60°C for 4min) × 26 cycles/4°C hold until ready to purify
	dhfr-M3	5′-TGATGGAACAAGTCTGCGACGTT-3′		
*Pfdhps*				
PCR1	dhps-N1	5′-GATTCTTTTTCAGATGGAGG-3′	770	94°C for 3min/(94°C for 30s- 55°C for 30s- 65°C for 60s) × 30cycles / 65°C for 5min/ 15°C for 5min.
	dhps-N2	5′-TTCCTCATGTAATTCATCTGA-3′	711	
Nested	dhps-R2	5′-AACCTAAACGTGCTGTTCAA-3′		94°C for 5min/(94°C for 30s- 60°C for 30s- 65°C for 1min) × 30cycles /65°C for 5min/15°C for 5min.
	dhps-R/	5′-AATTGTGTGATTTGTCCACAA-3′		
	dhps-R2	5′-AACCTAAACGTGCTGTTCAA-3′		96°C for 1min/(96°C for 30s-50°C-60°C for 4min) × 26 cycles/ 4°C hold until ready to purify.
Sequencing	dhps-R/	5′-AATTGTGTGATTTGTCCACAA-3′		
*Pfmdr1*				
PCR1	fn1/1	5′-ACAAAAAGAGTACCGCTGAAT-3′	578	94°C for 3min/(94°C for 30s- 55°C for 30s- 65°C for 1min) × 30cycles/ 65°C for 5min/ 15°C for 5min.
	rev/c1	5′-AAACGCAAGTAATACATAAAGTC-3′	534	
Nested	mdr2/1	5′-ACAAAAAGAGTACCGCTGAAT-3′		94°C for 3min/(94°C for 30s- 60°C for 30s- 65°C for 1min) × 30cycles/ 65°C for 5min/ 15°C for 5min.
	newrev1	5′-AAACGCAAGTAATACATAAAGTC-3′		
Sequencing	mdrfr1f-seq	5′-GTCGAATTATTTAGAAAAAT-3′		96°C for 1min/(96°C for 30s- 50°C -60°C for 4min) × 26cycles/4°C hold until ready to purify.
	mdrfr1r-seq	5′-GCAAGTAATACATAAAGT-3′		
*Pfcrt*				
	Crtd1	5′-TGTGCTCATGTGTTTAAACTT 5′-	166	94°C for 3min/(94°C for 30s- 55°C for 30s- 65°C for 1min) × 30 cycles/ 65°C for 5min/15°C for 5min.
	Crtd2	CAAAACTATAGTTACCAATTTTG		

### Statistical methods

For each mutation, are estimated the proportion, p_1_, of genotyped samples that were positive for the mutation, and its standard error, taking account of the different survey design used in each year using the survey commands in Stata 12 (StataCorp, College Station, Texas). The prevalence of parasitaemia p_2_ are also estimated, and its standard error. The prevalence of the different mutations in the population were then estimated, based on the prevalence of the mutation among genotyped samples, and the overall prevalence of parasitaemia, i.e. as p = p_1_ x p_2_, the standard error for log(p) obtained by the delta method was used to calculate 95% confidence interval. Comparisons between intervention and control areas were made by calculating the prevalence ratio, again using the delta method for calculating standard errors. Due to secular changes in prevalence of mutations in the study area over time the year of sampling could act as a confounder of the association between SMC and the prevalence of mutations. Consequently, when comparing prevalence of mutations over the entire study period (shown in Table 5), are accounted for the fact that more health posts implemented SMC later in the study (9/54 in 2008, 27/54 in 2009, 45/54 in 2010). By adjusting for study year in the binomial regression model used to estimate the prevalence ratios.

**Table 2:** Prevalence of resistance mutations in 2008 among those typed and estimated prevalence in the population

	**Study samples**		**Prevalence in population**^**$**^			
	**Control area**	**SMC area**	**Control area**	**SMC area**	**Prevalence ratio^**	**p-value**
					**SMC/ non SMC (95% CI)**	
Prevalence of parasitaemia:	66/1686	18/1019	4.86	1.46	0.30 (0.15, 0.61)	0.001
Prevalence of mutations						
dhfr triple (51, 59, 108)	27/37	7/8	2.88	1.55	0.54 (0.25, 1.17)	0.12
dhps-437	25/37	7/8	2.68	1.55	0.58 (0.25, 1.34)	0.20
SP resistant mutant (dhfr triple + dhps 437)	23/37	6/8	2.47	1.32	0.54 (0.22, 1.30)	0.17
crt CVIET mutation	8/35	2/3	0.87	1.18	1.36 (0.39, 4.71)	0.63
mdr 86Y	0/19	1/5	0	0.35	-	-
mdr 184F	13/19	4/5	2.74	1.41	0.52 (0.23, 1.14)	0.10
AQ resistant mutant (mdr 86Y + crt CVIET)	0/13	1/2	0	0.88	-	-
SP resistant & AQ resistant	0/11	1/2	0	0.88	-	-

^ Prevalence ratios accounting for survey design. $For genotypes, prevalence among population is estimated as the product of 1) the probability of a resistant genotype among the typed samples and 2) the probability positive among samples with a definitive result (positive or negative) for asexual stage parasitaemia. Standard errors estimated by the delta method as described in the methods section.

## Results

Of the 2,721 children surveyed in 2008, blood samples were obtained from 2,705 children. 84 were positive for *P. falciparum* after microscopic examination of slides, 18/1,019 (1.8%) in the SMC area and 66/1,686 (3.9%) in the control area (survey-adjusted prevalence ratio 0.30 (95% CI: 0.15, 0.61), p=0.001). In 2009, 6809 children were surveyed, samples were obtained from 6,646, of which 50 had positive blood films, 9/3,326 (0.27%) and 41/3320 (1.2%) respectively in the SMC and control areas; aPR 0.16 (0.06, 0.42), p<0.001. In 2010, 1,098 were surveyed, samples were obtained for 1,098 children, of which 27 were positive by microscopy, 21/882 (2.4%) and 6/216 (2.8%) respectively in the SMC and control areas, aPR 0.71 (0.23, 2.20), p=0.55.

### *Pfdhfr* polymorphisms

In 2008, 45 of 84 samples, all samples (43 and 27) in 2009 and 2010 were positive for *P. falciparum* by PCR. The prevalence of IRNI triple-mutant haplotypes of *pfdhfr* in 2008 stood at 7/8 (87.5%) in SMC villages *versus* 27/37 (72.9%) in control villages (survey-adjusted p-value = 0.002). In 2009, the prevalence was 4/6 (66.6%) in intervention villages and 27/34 (79.4%) in control villages (p = 0.50). In 2010, the prevalence of IRNI triple-mutant was 19/21 (90.4%) and 4/4 (100%) in SMC villages and control villages respectively (p = 0.46). The allele I164L mutation was not found among Senegalese isolates (Tables 2, 3, 4).

**Table 3:** Prevalence of resistance mutations in 2009 among those typed and estimated prevalence in the population

	**Study samples**		**Prevalence in population**^**$**^			
	**Control area**	**SMC area**	**Control area (%)**	**SMC area (%)**	**Prevalence ratio^**	**p-value**
					**SMC/ non SMC (95% CI)**	
Prevalence of parasitaemia among children surveyed	41/3320	9/3326	1.33	0.22	0.16 (0.06, 0.42)	<0.001
Prevalence of mutations among typed						
dhfr triple (51, 59, 108)	27/34	4/6	0.98	0.18	0.18 (0.059, 0.58)	0.004
dhps-437	25/35	4/6	0.88	0.18	0.21 (0.064, 0.66)	0.008
SP resistant mutant (dhfr triple + dhps 437)	23/34	4/6	0.84	0.18	0.22 (0.068, 0.69)	0.01
crt CVIET mutation	13/35	3/6	0.46	0.14	0.30 (0.10, 0.86)	0.026
mdr 86Y	15/33	4/6	0.56	0.18	0.32 (0.11, 0.92)	0.034
mdr 184F	28/34	5/6	1.02	0.23	0.22 (0.086, 0.58)	0.002
AQ resistant mutant (mdr 86Y + crt CVIET)	9/33	2/6	0.34	0.09	0.27 (0.078, 0.92)	0.036
SP resistant & AQ resistant	8/32	2/6	0.31	0.09	0.29 (0.08, 1.07)	0.064

^ Prevalence ratios accounting for survey design. $For genotypes, prevalence among population is estimated as the product of 1) the probability of a resistant genotype among the typed samples and 2) the probability positive among samples with a definitive result (positive or negative) for asexual stage parasitaemia. Standard errors estimated by the delta method as described in the methods section.

**Table 4:** Prevalence of resistance mutations in 2010 among those typed and estimated prevalence in the population

	**Study samples**		**Prevalence in population**^**$**^			
	**Control area**	**SMC area**	**Control area**	**SMC area**	**Prevalence ratio^**	**p-value**
					**SMC/ non SMC (95% CI)**	
Prevalence of parasitaemia:	6/216	21/882	2.47	1.76	0.71 (0.23, 2.20)	0.55
Prevalence of mutations						
dhfr triple (51, 59, 108)	4/4	19/21	2.78	2.15	0.78 (0.30, 1.98)	0.60
dhps-437	4/4	18/21	2.78	2.04	0.74 (0.28, 1.90)	0.52
SP resistant mutant (dhfr triple + dhps 437)	4/4	18/21	2.78	2.04	0.74 (0.28, 1.90)	0.52
crt CVIET mutation	2/5	8/22	1.11	0.87	0.78 (0.15, 4.09)	0.77
mdr 86Y	0/5	12/22	0	1.30	-	-
mdr 184F	2/5	15/22	1.11	1.62	1.46 (0.27, 8.05)	0.66
AQ resistant mutant (mdr 86Y + crt CVIET)	0/5	5/22	0	0.54	-	-
SP resistant & AQ resistant	0/4	5/21	0	0.57	-	-

^ Prevalence ratios accounting for survey design. $For genotypes, prevalence among population is estimated as the product of 1) the probability of a resistant genotype among the typed samples and 2) the probability positive among samples with a definitive result (positive or negative) for asexual stage parasitaemia. Standard errors estimated by the delta method as described in the methods section.

**Table 5:** Prevalence of resistance mutations among children with parasitaemia pooled over the entire study

**Mutation**	**Non-SMC area n positive / N typed (%)**	**SMC area n positive / N typed (%)**	**Crude prevalence ratio SMC/ non SMC (95% CI)**	Adjusted prevalence ratio* SMC/ non SMC (95% CI)	**p-value**
dhfr triple (51, 59, 108)	59/76 (77.6)	29/34 (85.3)	1.10 (0.91, 1.32)	0.90 (0.79, 1.02)	0.087
dhps-437	55/77 (71.4)	28/34 (82.4)	1.15 (0.93, 1.42)	0.98 (0.80, 1.21)	0.867
SP resistant mutant (dhfr triple + dhps 437)	51/76 (67.1)	27/34 (79.4)	1.18 (0.94, 1.49)	0.89 (0.75, 1.06)	0.182
crt CVIET mutation	23/76 (30.3)	12/30 (40.0)	1.32 (0.76, 2.31)	1.46 (0.70, 3.05)	0.311
mdr 86Y	15/58 (25.9)	16/32 (50.0)	1.93 (1.1, 3.38)	1.86 (1.04, 3.34)	0.037
mdr 184F	44/59 (74.6)	23/32 (71.9)	0.96 (0.74, 1.26)	1.09 (0.82, 1.47)	0.550
AQ resistant mutant (mdr 86Y + crt CVIET)	9/52 (17.3)	7/29 (24.1)	1.39 (0.58, 3.37)	1.90 (0.71, 5.09)	0.205
SP-AQ resistant	8/48 (16.7)	7/28 (25.0)	1.50 (0.61, 3.72)	2.04 (0.73, 5.67)	0.173

Prevalence ratios from binomial regression model with robust standard errors *Adjusted for year of the study as described in the statistical methods.

### *Pfdhps* polymorphisms

All the 2008, 2009 and 2010 samples were wild-type for the 540 codon of *pfdhps*. The prevalence of mutation of *pfdhps* 437G allele was 7/8 (87.5%) in SMC villages *versus* 25/37 (67.56%) in control villages during the first year (p = 0.50). After two and three years of implementation, prevalence was 4/6 (66.6%) and 18/21 (85.7%) in SMC villages and 25/35 (71.4) and 4/4 (100%) in control villages, respectively (p = 0.88 and p = 0.48 respectively). The *pfdhfr/pfdhps* IRNI/GK quadruple mutant was 6/8 (75%), 4/6 (66.6%) and 18/21 (85.7%) in SMC villages and 23/37 (62.1), 23/34 (67.6%) and 4/4 (100%) in control villages in 2008, 2009 and 2010, respectively (all p-values > 0.2).

### *Pfcrt* polymorphisms

The *pfcrt* haplotypes CVIET and CVMNK were successfully identified in 38 samples in 2008, 41 samples in 2009 and 27 samples in 2010. Prevalence of CVIET haplotypes in intervention villages was 2/3 (66.6%) in 2008, versus 8/35 (22.8%) in control villages (p = 0.038). It was 3/6 (50%) and 13/35 (37.1%), respectively, in SMC villages and control villages in 2009 (p=0.55). In 2010 the CVIET haplotypes was found in 8/22 (36.3%) of positive samples in SMC villages versus 2/5 (40%) in control villages (p=0.72) (Tables 2, 3, 4).

### *Pfmdr1* polymorphisms

Twenty-four samples from 2008, 40 samples from 2009 and 27 isolates from 2010 were successfully genotyped for *pfmdr1* at codon 86 and 184. In 2008, prevalence of the mutation pfmdr1-86Y allele was 1/5 (20%) in SMC villages, with no mutation was found at codon 86 in control villages (p=0.066). In 2009, the prevalence was 4/6 (66.6%) in SMC villages and 15/33 (45.4%) in control villages (p=0.03). In 2010, the 86Y mutation was observed in 12/22 (54.5%) and no mutation in control villages (p=0.051). During the first year, mutant 184F alleles were found in 4/5 (80) and 13/19 (68.4) SMC and control villages, respectively (p=0.33). In 2009, prevalence of this mutation was 5/6 (83.3) and 28/34 in SMC and control villages (p=0.74). In 2010, this was 15/22 (68.1) and 2/5 in 2010, (p=0.56) (Tables 2, 3, 4).

## Discussion

Malaria remains a major concern in Senegal, despite the significant reduction in disease incidence observed in parts of the country in recent years (according to data of the National Malaria Control Programme of Senegal). It has become urgent to find a new strategy for the prevention of malaria especially among children less than ten years residing in areas of seasonal malaria, for which the risk of dying from malaria remains high. SMC with SP+AQ is now recommended by WHO for the control of malaria in areas of the Sahel and sub-Sahel with high seasonal malaria transmission. However, the long-term sustainability of SMC will depend on sensitivity of parasites to the anti-malarial drugs used. In this study, the impact of the use of SP+AQ over the course of three years are measured, on an increasingly large scale, on the prevalence of molecular markers of resistance in characterizing the prevalence of mutation in *pfdhfr* and *pfdhps* genes*,* associated with resistance to SP, and genes *pfmdr1* and *pfcrt*, markers of resistance to CQ and AQ.

Among children with parasitaemia at the end of the transmission season, the prevalence of mutation *pfmdr1*-86Y was higher among children that had received SMC. There was no strong evidence that the prevalence of other mutations, among infected children, was different in SMC and control areas. However, despite the large sample size of these surveys, the number of positives was low and confidence intervals were wide due to reduced transmission during the study period.

In each year, the overall prevalence of SP-resistant genotypes was lower in SMC areas, as a consequence of the marked reduction in prevalence of infection in areas using SMC. For *pfcrt*-CVIET in 2008 and *pfmdr1*-184F in 2010, point estimates of the prevalence ratio were above 1, but in both cases the confidence in intervals were wide.

Differences in the absolute prevalence of resistance genotypes are seen most clearly in 2009, the year when there were an equal number of clusters in the intervention and control groups.

The presence of mutations at codons 540 of the *pfdhps* gene and 164 of the *pfdhfr* gene was not detected in this study. These mutations are considered as confirmed markers of resistance to SP, and appear associated with treatment failure in East Africa [13]. The high prevalence of the *pfdhfr* triple mutation (88/110 samples typed) and the *pfdhfr* / *pfdhps* quadruple mutation (83/111 samples) observed in this study is not very different from the results observed after two years of implementation of SMC with SP in infants in Senegal [7]. This is consistent with the results obtained in Gabon, in Senegal and in Cameroon [14-16].

The quadruple mutation was observed in 83/111 samples. Intergenic association of *pfdhfr* and pfdhps mutant codons was found in other studies where SP resistance was found to be associated with double up to quintuple mutations in both genes [17,18].

The prevalence of *pfcrt* CVIET among children positive at the end of the transmission season (10/38 in 2008, 16/41 in 2009 and 10/27 in 2010) is low compared to previous estimates; the prevalence of *pfcrt* mutation between 2004 and 2006 was about 60% in Senegal and Kenya [19,20]. This may be due to the fact that in Senegal, CQ has been abandoned as first-line drug treatment against malaria since 2002, first replaced with SP + AQ and from 2006 onwards with ACT. A substantial decrease in the prevalence of mutations at the level of the *pfcrt* gene several years after discontinuation of CQ as the first-line drug was observed in Malawi by Kublin *et al.*[21] and Mita *et al.*[22]. Over three years of SMC, the results showed that the prevalence of *pfcrt* and *pfmdr1* mutation was more frequent in SMC areas but the only significant difference was for *pfmdr1*-86Y. The association mutations *pfcrt*, *pfmdr1*, *pfdhfr* and *pfdhps* were demonstrated by authors in West Africa and in Angola [23-25]. Almost all isolates carrying the mutant genotype for *pfdhfr* or *pfdhps* (conferring resistance to SP), carry at least one of the mutations associated with AQ (*pfcrt* and or *pfmdr1*).

However, despite the high rates of mutations observed at these genes, SP and/or AQ remain effective for the treatment and prevention of malaria in West Africa [15,26-28]. The value of specific combinations of these molecular markers for the predicting efficacy of SMC needs to be established in settings with different levels of malaria transmission and acquired immunity [29]. However, defining a resistance threshold for acceptable efficacy may be problematic given that each SMC study will contribute only a single data point to examine this relationship, as was the case for studies of IPTi [30]. A limitation of this study is that prevalence of resistant markers was measured only in the target age group for SMC, and may not indicate changes in prevalence of the different resistance genotypes in the whole population.

The prevalence of different resistance genotypes at the end of the transmission season may simply represent differential survival of resistant parasites, which is necessary but not sufficient for SMC to lead to increased transmission of drug resistant genotypes. Also relevant will be the relative importance of children under five as a source of onward transmission in relation to adults and older children. Adults and older children will not receive SP+AQ for SMC and should not receive SP or AQ for treatment of symptomatic malaria where SMC is deployed. Consequently, because prevalence among children treated with SMC is much lower than among children who do not receive SMC, the fraction of the total population with SP and AQ resistant parasites will be lower than if SMC is not used. Monitoring among older children or adults was not undertaken in this study, but should be used as a means to monitor population-level changes in the prevalence of drug resistance markers in the future. Molecular analysis using DNA extracted from rapid diagnostic tests [31,32] would be one practical approach to measure these changes.

## Conclusion

The *dhfr-dhps* quintuple mutation was not observed in either SMC or control health posts after three years of SMC implementation in the study area. Analysis of individual mutations showed that *pfmdr1*-86Y was more common among children positive for parasites at the end of the transmission season in SMC areas. However, the absolute prevalence of resistance markers was lower in SMC areas, reflecting the reduction in prevalence due to the intervention, and this is not expected to compromise the efficacy of regimens used for case management. Evaluation of the prevalence of markers of drug resistance should be part of routine monitoring and evaluation in areas where SMC is deployed.

## Competing interests

The authors declared that they have no competing interests concerning the work reported in this paper.

## Authors’ contributions

BC, BF and PM conceived and designed the study. AL was responsible of the molecular analysis and genotyping. MC and PM analysed the data. AL drafted the manuscript. All authors read and approved the final manuscript.
